# National Institutes of Health Funding Gaps for Principal Investigators

**DOI:** 10.1001/jamanetworkopen.2023.31905

**Published:** 2023-09-19

**Authors:** Kelly M. Gillen, Daniel M. Markowitz, Patricia Long, Adriel Villegas-Estrada, Eileen Chang, Ajay Gupta

**Affiliations:** 1 Department of Radiology, Weill Cornell Medicine, New York, New York

## Abstract

**Importance:**

Early-stage and established investigators compete for a limited supply of funds from the National Institutes of Health (NIH). Regardless of their previous funding success, many principal investigators (PIs) encounter a funding gap in which they no longer receive ongoing funding from the NIH.

**Objective:**

To determine incidence rates of PI-level funding gaps, the mean funding gap length, and whether these 2 metrics are associated with previous funding success.

**Design, Setting, and Participants:**

This study was conducted using data from NIH RePORTER. Historical datafiles for fiscal year (FY) 2011 to FY 2021 were aggregated to generate 2 master datafiles for this period: all NIH awards and only R01 awards. PIs with no funding in FY 2011 or FY 2021 were removed. PIs were sorted by FY 2011 total funding amounts and grouped by quarter of amount.

**Results:**

A total of 39 944 unique researchers were awarded 220 131 NIH awards, of which 103 753 were R01 awards. For all NIH awards, there was an overall linear increase from top quarter to bottom quarter in the percentage of PIs who had at least 1 year without funding (from 27% to 75%), percentage of these gap PIs who had at least 2 consecutive years without funding (from 56% to 68%), and mean maximum consecutive years without funding for gap PIs (2.2 years to 3.1 years). For only R01 awards, there was an overall linear increase from top quarter to bottom quarter in the percentage of PIs who had at least 1 year without funding (50% to 74%), percentage of gap PIs who had at least 2 consecutive years without funding (59% to 71%), and mean maximum consecutive years without funding for gap PIs (2.4 years to 3.1 years).

**Conclusions and Relevance:**

In this cohort study of NIH-funded investigators, PIs with higher NIH funding were less likely to experience a funding gap. Additionally, when these PIs encountered a funding gap, this period without funding was shorter; however, among all PIs, funding gaps typically lasted 2 to 3 years. These associations were found inclusive of all NIH awards and when analysis was limited to only R01 awards. These findings may be useful to PIs and academic institutions as they prepare, structure, and project research resource allocations.

## Key Points

**Question:**

What percentage of principal investigators (PIs) encounter a funding gap, what is the mean funding gap length, and do these 2 metrics correlate with previous funding success?

**Findings:**

In this cohort study of 39 944 researchers who received 220 131 National Institutes of Health (NIH) awards, PIs with higher amounts of NIH funding were less likely to experience a funding gap and any funding gap they encountered was shorter vs those for PIs with other funding amounts. Among all PIs, funding gaps typically lasted 2 to 3 years.

**Meaning:**

This analysis may be useful to PIs and academic institutions as they prepare, structure, and project research resource allocations, working with investigators to develop a strategy for offsetting the risks and consequences of encountering a funding gap.

## Teaser

This cohort study examines data for research funding to determine the length of any funding gaps at the level of principal investigator and whether these gaps are associated with the investigator’s previous funding success.

## Introduction

Early-stage and established investigators compete for a limited supply of funds from the National Institutes of Health (NIH). Despite the allocation of more than $33 billion across 58 368 awards in fiscal year (FY) 2022 alone, obtaining and sustaining NIH funding throughout the career of a principal investigator (PI) remains a formidable challenge.^1^ Ongoing initiatives aim to address inequalities in funding based on career stage and other demographic characteristics of the scientific workforce, but recent historical data show that 10% of research project grant funding is awarded to the top 1% of PIs.^2^ A potential consequence of this concentrated funding is that 72.7%, 62.4%, and 63.7% of early-, middle-, and late-stage investigators, respectively, are supported by just 1 research project grant.^3^ This indicates a heavy reliance on a single award and, consequently, the PI’s ability to successfully obtain a new or renewal award. With a new research project grant funding success rate of 20.7%, many previously funded researchers encounter a funding gap when their NIH funding has concluded, and they receive no ongoing funding from the NIH as an research project grant PI.^1^

Understanding PI-level funding gap trends may be of value to PIs and academic institutions as they prepare, structure, and project research resource allocations; this NIH funding is often paramount to both the success of a researcher and that of their academic institution. Therefore, the purpose of this study was to use NIH RePORTER data to determine funding gap incidence rates, the mean funding gap length, and whether these 2 metrics are associated with previous funding success.

## Methods

### Data Sources

All data were collected from the Research Portfolio Online Reporting Tools of NIH RePORTER.^4^ The NIH has released annual datafiles for FY 1985 to FY 2021. Historical datafiles for FY 2011 to FY 2021 were aggregated to generate 2 master datafiles for this period: all NIH awards (n = 220 131) and only R01 awards (n = 103 753). All unique researchers (n = 39 944) were identified. For consistency, all researchers and their respective funding histories were tracked using NIH person ID rather than name. Linking an award to a researcher was only done if a researcher was listed as a PI. Per title 45 of the Code of Federal Regulations,^5^ this research is exempt from institutional review board approval as secondary research for which consent is not required because it uses identifiable private information that is publicly available. All awards and funding data in this study were limited to clinical departments as categorized by the Blue Ridge Institute for Medical Research: anesthesiology, dermatology, emergency medicine, family medicine, internal medicine/medicine, neurology, neurosurgery, obstetrics and gynecology, ophthalmology, orthopedics, otolaryngology, pathology, pediatrics, physical medicine and rehabilitation, plastic surgery, psychiatry, public health and preventive medicine, radiation-diagnostic/oncology, surgery, urology.^6^

### Statistical Analysis

Funding gaps at the PI level from FY 2011 to FY 2021 were investigated. First, data for PIs with no funding in FY 2011 or FY 2021 were removed. Then, to analyze PIs by funding tier, researchers were sorted by FY 2011 total funding amounts and grouped by quarter of funding amount (ie, top quarter, middle-top quarter, middle-bottom quarter, bottom quarter).

The FY 2011 distribution of award types by quarter for this cohort of NIH-funded investigators was first investigated.^7^ Next, the maximum consecutive years without funding for each PI was calculated. For each quarter, the total number of researchers in each consecutive years without funding category was counted (ie, zero funding gaps to 9 consecutive years without funding). Lastly, and again grouped by quarter, the following statistical outputs were generated: FY 2011 mean number of awards per PI, FY 2011 mean number of R01 awards per PI, number of PIs who had a funding gap of at least 1 year, number of PIs who had a funding gap of at least 2 consecutive years, and mean maximum consecutive time in years without funding for all PIs with a funding gap of at least 1 year. This analysis was performed inclusive of all NIH awards and then repeated for R01 awards only.

The data were aggregated, sorted, and analyzed using Microsoft Excel for Microsoft 365 (version 2202). Standard functions and formulas were used, including counts, sums, means, standard deviations, and charts. All statistical tests were performed using GraphPad Prism (version 9.5.1.733). To assess the statistical significance of differences with categorical variables, a χ^2^ test of independence was performed with a significance threshold of *P* < .05. For the statistical comparison of continuous variables between 3 or more groups, a Kruskal-Wallis test was performed with a significance threshold of *P* < .05. If significance was determined in the Kruskal-Wallis test, a post hoc multiple comparison test was conducted with a Dunn test.

## Results

### All NIH Awards

**Figure 1.**
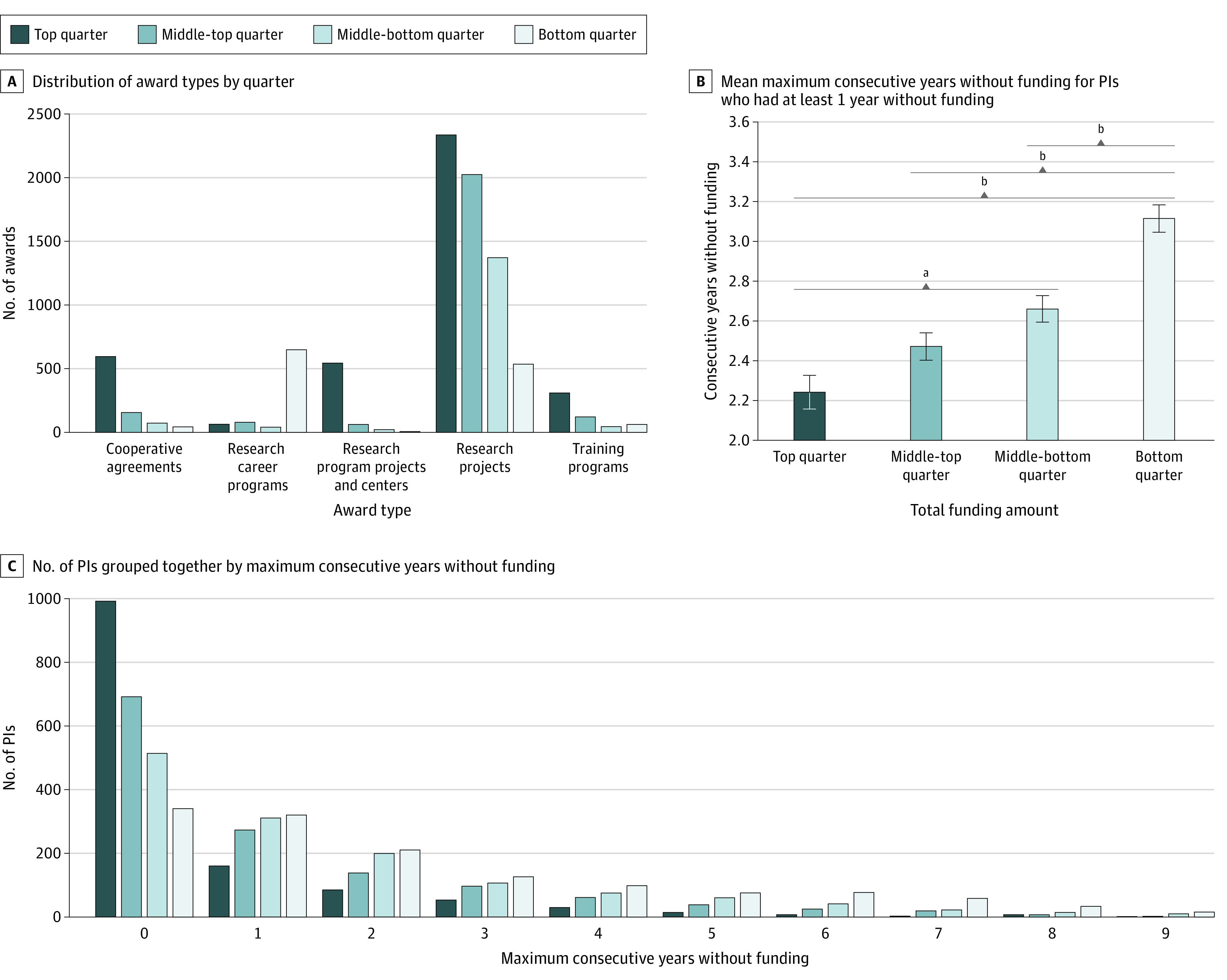
All National Institutes of Health (NIH) Awards From Fiscal Year (FY) 2011 to FY 2021 Quarters are assigned by the total NIH funding for each principal investigator (PI) in FY 2011. The top quarter of amounts range from $74 809 965 to $917 276; middle-top quarter, $916 847 to $477 387; middle-bottom quarter, $477 007 to $282 475; and bottom quarter, $282 000 to $2539. The distribution of award types by quarter (A) shows the number of awards linked to PIs in FY 2011; each quarter represents 1352 PIs. Mean number of maximum consecutive years (B) without funding is shown for PIs who had at least 1 year without funding (gap PIs). Error bars indicate SE. The top quarter represents 360 PIs; middle-top quarter, n = 660; middle-bottom quarter, n = 838; and bottom quarter, n = 1012. In the number of PIs grouped by maximum consecutive years without funding (C), each quarter represents 1352 PIs. Statistical significance is calculated by a Kruskal-Wallis test with a post hoc Dunn multiple comparisons test. ^a^*P* = .009. ^b^*P* < .001.

A total of 39 944 unique researchers were awarded 220 131 NIH awards. The FY 2011 distribution of award types by quarter of funding amount is shown in Figure 1A. Ninety-eight percent of all awards linked to PIs in FY 2011 fell into the following 5 categories: cooperative agreements (U series), research career programs (K series), research program projects and centers (P series), research projects (R series), and training programs (T series). While the top quarter had 2335 of 6266 research projects (37%), it had 596 of 869 cooperative agreements (69%) and 544 of 635 research programs projects and centers (86%).^7^

Figure 1C shows the distribution of PIs by maximum consecutive years without funding. Among top-quarter PIs, 992 of 1352 (73%) received continuous funding from FY 2011 to FY 2021; however, only 340 of 1352 bottom-quarter PIs (25%) received continuous funding over this same period. The funding gap incidence rates and the mean funding gap length are presented by quarter in Table 1, along with the FY 2011 mean number of awards and mean number of R01 awards per PI. There was an overall linear increase from top quarter to bottom quarter in the percentage of PIs who had at least 1 year without funding (27% to 75%), percentage of gap PIs who had at least 2 consecutive years without funding (56% to 68%) (Table 1), and mean maximum consecutive years without funding for gap PIs (2.2 years to 3.1 years) (Figure 1B).

**Table 1.** All National Institutes of Health Awards (FY 2011 to FY 2021): Funding Gap Analysis Summary Statistics^a^

Metric	No./total No. (%)			
	Top quarter	Middle-top quarter	Middle-bottom quarter	Bottom quarter
FY 2011 mean No. of awards per PI	3.1	1.9	1.2	1.1
FY 2011 mean No. of R01 awards per PI	1.5	1.3	0.9	0.1
PIs with funding gap of ≥1 y	360/1352 (27)	660/1352 (49)	838/1352 (62)	1012/1352 (75)
Gap PIs with funding gap of ≥2 consecutive years	200/360 (56)	387/660 (59)	527/838 (63)	692/1012 (68)
Gap PIs maximum consecutive time without funding, mean, y	2.2	2.5	2.7	3.1

Abbreviations: FY, fiscal year; gap PI, principal investigator who had ≥1 year without funding; PI, principal investigator; R01, research project.

^a^ Quarters are assigned by total National Institutes of Health funding amount for each PI in FY 2011.

Furthermore, there was a significant association between quarter and percentage of PIs who had at least 1 year without funding (χ^2^_3_ = 690.1; n = 5408; *P* < .001) and quarter and percentage of gap PIs who had at least 2 consecutive years without funding, (χ^2^_3_ = 26.49; n = 2870; *P* < .001). As shown in Figure 1B, a Kruskal-Wallis test on the mean maximum consecutive years without funding for gap PIs yielded significant variation among quarters (H_3_ = 56.64; *P* < .001). A post hoc Dunn multiple comparisons test showed that 1 of 6 comparisons differed significantly at *P* = .009 and 3 of 6 comparisons differed significantly at *P* < .001.

### Only R01 Awards

**Figure 2.**
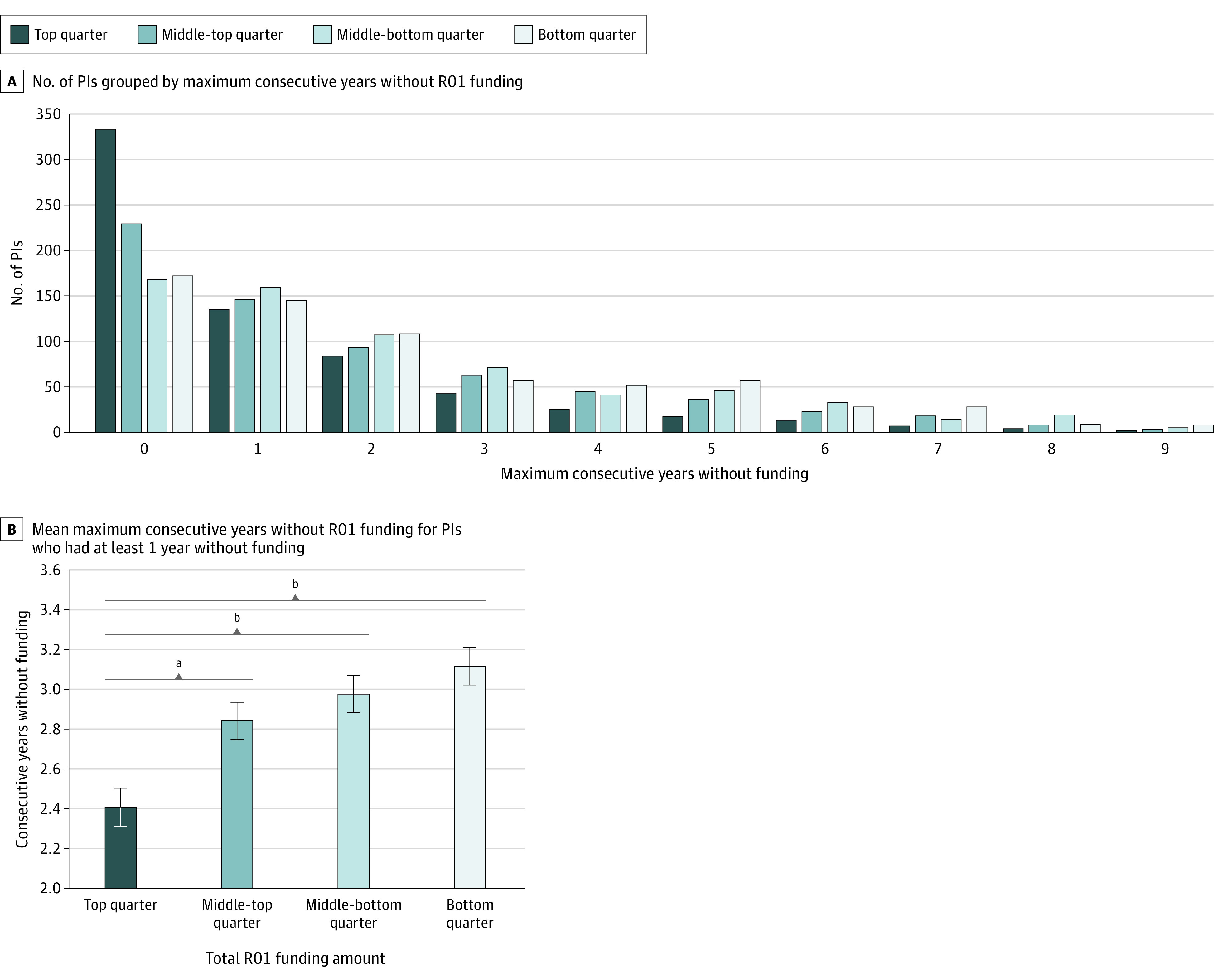
Only Research Project (R01) Awards From Fiscal Year (FY) 2011 to FY 2021 Quarters are assigned by the total R01 funding for each principal investigator (PI) in FY 2011. The top quarter of amounts range from $3 828 698 to $728 925; middle-top quarter, $728 449 to $460 315; middle-bottom quarter, $460 221 to $341 902; and bottom quarter, $341 842 to $2539. In the number of PIs grouped by maximum consecutive years without funding (A), the top quarter represents 663 PIs; middle-top quarter: n = 664; middle-bottom quarter: n = 663; and bottom quarter: n = 664. In the mean maximum consecutive years (B) without funding for PIs who had at least 1 year without funding (gap PIs), the top quarter represents 330 PIs; middle-top quarter, n = 435; middle-bottom quarter, n = 495; and bottom quarter, n = 492. Error bars indicate SE. Statistical significance is calculated by a Kruskal-Wallis test with a post hoc Dunn multiple comparisons test. ^a^*P* = .01. ^b^*P* < .001.

A total of 103 753 R01 awards were included in the analysis. Figure 2A shows the distribution of PIs by maximum consecutive years without funding. Among top-quarter PIs, 333 of 663 (50%) received continuous funding from FY 2011 to FY 2021; however, only 172 of 664 bottom-quarter PIs (26%) received continuous funding over this same period. The funding gap incidence rates and mean funding gap length are presented by quarter in Table 2, along with the FY 2011 mean number of R01 awards per PI. There was an overall linear increase from top quarter to bottom quarter in the percentage of PIs who had at least 1 year without funding (50% to 74%), percentage of gap PIs who had at least 2 consecutive years without funding (59% to 71%) (Table 2), and mean maximum consecutive years without funding for gap PIs (2.4 years to 3.1 years) (Figure 2B).

**Table 2.** Only R01 Awards (FY 2011 to FY 2021): Funding Gap Analysis Summary Statistics^a^

Metric	No./total No. (%)			
	Top quarter	Middle-top quarter	Middle-bottom quarter	Bottom quarter
FY 2011 mean No. of R01 awards per PI	2.4	1.5	1.0	1.0
PIs with funding gap of ≥1 y	330/663 (50)	435/664 (66)	495/663 (75)	492/664 (74)
Gap PIs with funding gap of ≥2 consecutive years	195/330 (59)	289/435 (66)	336/495 (68)	347/492 (71)
Gap PIs maximum consecutive time without funding, mean, y	2.4	2.8	3.0	3.1

Abbreviations: FY, fiscal year; gap PI, principal investigator who had ≥1 year without funding; PI, principal investigator; R01, research project.

^a^ Quarters are assigned by the total R01 funding amount for each PI in FY 2011.

Furthermore, there was a significant association between quarter and the percentage of PIs who had at least 1 year without funding (χ^2^_3_ = 119.4; n = 2654; *P* < .001) and quarter and the percentage of gap PIs who had at least 2 consecutive years without funding (χ^2^_3_ = 12.15; n = 1752; *P* = .007). As shown in Figure 2B, a Kruskal-Wallis test on the mean maximum connective years without funding for gap PIs yielded significant variation among quarters (H_3_ = 25.13; *P* < .001). A post hoc Dunn multiple comparisons test showed that 1 of 6 comparisons differed significantly at *P* = .01 and 2 of 6 comparisons differed significantly at *P* < .001.

## Discussion

We analyzed historical funding gaps at the individual researcher level across all clinical departments over a 10-year period. In this cohort study, we found that PIs with higher NIH funding were less likely to experience a funding gap. Additionally, when these PIs encountered a funding gap, this period without funding was shorter; however, among all PIs, funding gaps typically lasted 2 to 3 years. These associations were found inclusive of all NIH awards and when analysis was limited to only R01 awards.

Our analysis of the FY 2011 distribution of award types showed that the PIs with the top quarter of funding amounts held 69% of cooperative agreements (U series) and 86% of research program projects and centers (P series). These results are consistent with idea that PIs with greater total funding in a given year would likely have NIH awards associated with higher dollar amounts. By extension, such PIs may use this type of funding success to generate subsequent research projects and reduce the probability of encountering a funding gap.

The NIH has produced numerous studies looking at funding rates for PIs at different career stages. Specifically, one study presented data on R01-equivalent funding dynamics (eg, R01, R23, R29, R37, R56, RF1, DP2, DP1, DP5, RL1, and U01) for at-risk investigators (ie, PIs who have submitted applications near the end of a funding cycle and for whom a funding gap would occur if the applications under consideration were not awarded).^8^ While funding rates for at-risk investigators have shown recent improvement, they still lag behind funding rates for established investigators (ie, researchers who are assured of continuous funding for at least 1 more fiscal year).^8^ For example, in FY 2021, the funding rate for at-risk investigators decreased to 25% while the funding rate for established investigators remained at 31%.^9^

A recent NIH advisory committee recommended that at a programmatic level, meritoriously scored applications from both early-stage and at-risk investigators be given additional consideration and prioritized for funding.^10^ Our study included at-risk investigators, but we extended the analysis to further quantify and understand what happens to such investigators once they lose all funding, including how such funding gaps persist. Overall, we believe our study may be useful to PIs and academic institutions as they prepare, structure, and project research resource allocations. Administrators at academic medical centers and the NIH should work collaboratively with investigators, encouraging a proactive mindset and developing a strategy to offset the risks and undesirable consequences of encountering a funding gap.

One of the key results of our work was that funding gaps lasted a mean of 2 to 3 years, which is approximately half the time of a standard 5-year R01 project. An understanding of the mean time needed to recover from a funding gap may help investigators develop a grant submission timeline strategy that minimizes the likelihood of a funding gap. Furthermore, our results suggest the potential need for bridge-type funding options should a funding gap occur. Such a program, even if just 1 year in duration, could provide a mechanism to shorten or even prevent funding gaps.

### Limitations

There are several limitations to this study. Although the first portion of the analysis included all NIH awards, the second portion of the analysis was limited to R01 awards. In this way, we did not apply our research methodology to an R01-equivalent award data set as was used in the previously mentioned NIH studies on at-risk investigators. We were unable consider any PI-specific reasons for a funding gap. Perhaps a researcher secured funding from philanthropy, a private foundation, or another public source and therefore did not submit NIH grant applications for the corresponding period. Similarly, a physician scientist may have chosen to allocate several years to more clinical, patient-centered work, likely contributing to a block of time when NIH grant funding was less vital or even unnecessary. Additionally, we focused our analysis on clinical departments to strive for maximum applicability to PIs and administrators at academic medical centers. We recognize that our results could potentially change if we included all departments, not just the clinical ones, in our data set. Another limitation is the lack of demographic data available for each PI. This led to our inability to specifically explore any gender, racial, or ethnic disparities. Such analyses are critical to promoting and achieving diversity and equity in academic research.^11,12,13^

## Conclusions

This cohort study of NIH-funded investigators found that while the probability of a PI experiencing a funding gap varied between quarters, such funding gaps among all PIs typically lasted 2 to 3 years. Given the potential disruptions to a research program caused by such lengthy gaps, further investigation into strategies to mitigate the risk and impact of these funding gaps is warranted.

## Notes

Supplement.
Data Sharing Statement
