# Supplementary material for: National Institutes of Health Funding Gaps for Principal Investigators
Source: JAMA Netw Open. 2023 Sep 19;6(9):e2331905. doi: 10.1001/jamanetworkopen.2023.31905 (PMC10509726; doi:10.1001/jamanetworkopen.2023.31905)
Supplement: Supplement. — Data Sharing Statement [file jamanetwopen-e2331905-s001.pdf]

Data Sharing Statement

Gillen. National Institutes of Health Funding Gaps for Principal Investigators. *JAMA Netw Open*. Published September 12, 2023. doi:10.1001/jamanetworkopen.2023.31905

Data
Data available: Yes
Data types: Data (not involving human participants)
How to access data: keg2002@med.cornell.edu
When available: With publication

Supporting Documents
Document types: None

Additional Information
Who can access the data: Researchers whose proposed use of the data has been approved
Types of analyses: For any purpose
Mechanisms of data availability: With investigator support
